# Survival and New Prognosticators in Metastatic Seminoma: Results From the IGCCCG-Update Consortium

**DOI:** 10.1200/JCO.20.03292

**Published:** 2021-03-17

**Authors:** Jörg Beyer, Laurence Collette, Nicolas Sauvé, Gedske Daugaard, Darren R. Feldman, Torgrim Tandstad, Alexey Tryakin, Olof Stahl, Enrique Gonzalez-Billalabeitia, Ugo De Giorgi, Stéphane Culine, Ronald de Wit, Aaron R. Hansen, Marko Bebek, Angelika Terbuch, Costantine Albany, Marcus Hentrich, Jourik A. Gietema, Helene Negaard, Robert A. Huddart, Anja Lorch, Fay H. Cafferty, Daniel Y. C. Heng, Christopher J. Sweeney, Eric Winquist, Michal Chovanec, Christian Fankhauser, Daniel Stark, Peter Grimison, Andrea Necchi, Ben Tran, Axel Heidenreich, Jonathan Shamash, Cora N. Sternberg, David J. Vaughn, Ignacio Duran, Carsten Bokemeyer, Anna Patrikidou, Richard Cathomas, Samson Assele, Silke Gillessen

**Affiliations:** ^1^ Department of Medical Oncology, Inselspital, University Hospital, University of Bern, Bern, Switzerland; ^2^ European Organisation for Research and Treatment of Cancer, Brussels, Belgium; ^3^ Rigshospitalet, Copenhagen University Hospital, Copenhagen, Denmark; ^4^ Memorial Sloan Kettering Cancer Center, New York, NY; ^5^ Weill Medical College of Cornell University, New York, NY; ^6^ The Cancer Clinic, St Olavs University Hospital and Department of Clinical and Molecular Medicine, The Norwegian University of Science and Technology, Trondheim, Norway; ^7^ N.N. Blokhin Russian Cancer Research Center, Moscow, Russian Federation; ^8^ Research Institute of Oncology at Bashkir State Medical University, Ufa, Russian Federation; ^9^ Department of Oncology, Skåne University Hospital, Lund, Sweden; ^10^ Servicio de Oncologia Medica, Hospital Universitario 12 de Octubre, Madrid, Spain; ^11^ Universidad Catolica San Antonio de Murcia, UCAM, Murcia, Spain; ^12^ Istituto Scientifico Romagnolo per lo Studio e la Cura dei Tumori (IRST) IRCCS, Meldola, Italy and the Italian Germ Cell Cancer Group (IGG), Italy; ^13^ Department of Medical Oncology, Hôpital Saint-Louis, AP-HP, Faculté de Paris, Paris, France; ^14^ Erasmus MC Cancer Institute, Rotterdam, the Netherlands; ^15^ Division of Medical Oncology and Hematology, Princess Margaret Cancer Centre, University Health Network, Toronto, Ontario, Canada; ^16^ Department of Oncology, University Hospital Centre Zagreb, Zagreb, Croatia; ^17^ Division of Oncology, Department of Internal Medicine, Medical University of Graz, Graz, Austria; ^18^ Indiana University Melvin and Bren Simon Cancer Center, Indianapolis, IN; ^19^ Department of Hematology and Oncology, Red Cross Hospital, University of Munich, Munich, Germany; ^20^ University Medical Center Groningen, Groningen, the Netherlands; ^21^ Department of Oncology, Oslo University Hospital, Oslo, Norway; ^22^ Institute of Cancer Research, Sutton, United Kingdom; ^23^ Department of Medical Oncology and Hematology, University Hospital Zurich, Zurich, Switzerland; ^24^ Department of Urology, University Hospital Dusseldorf, Dusseldorf, Germany; ^25^ Medical Research Council Clinical Trials Unit at University College London (UCL), London, United Kingdom; ^26^ Tom Baker Cancer Centre, University of Calgary, Calgary, Alberta, Canada; ^27^ Department of Medical Oncology, Dana-Farber Cancer Institute, Boston, MA; ^28^ Division of Medical Oncology, Western University and London Health Sciences Centre, London, Ontario, Canada; ^29^ 2nd Department of Oncology, Faculty of Medicine, Comenius University and National Cancer Institute, Bratislava, Slovakia; ^30^ University of Zurich, Zurich, Switzerland; ^31^ Leeds Institute of Medical Research, University of Leeds, Leeds, United Kingdom; ^32^ Australian and New Zealand Urogenital and Prostate Cancer Trials Group, Sydney, Australia; ^33^ Fondazione IRCCS Istituto Nazionale dei Tumori, Milan, Italy. Current affiliation: Vita-Salute San Raffaele University and IRCCS San Raffaele Hospital and Scientific Institute, Milan, Italy; ^34^ Department of Medical Oncology, Peter MacCallum Cancer Centre, Melbourne, Australia; ^35^ Department of Urology, Uro-Oncology, Robot-Assisted and Specialized Urologic Surgery, University Hospital Cologne, Cologne, Germany; ^36^ St Bartholomew's Hospital, London, United Kingdom; ^37^ Medical Oncology, San Camillo Forlanini Hospital, Rome, Italy. Current affiliation: Englander Institute for Precision Medicine, Weill Cornell Medicine, New York-Presbyterian, New York, NY; ^38^ University of Pennsylvania, Philadelphia, PA; ^39^ Hospital Universitario Marques de Valdecilla and IDIVAL, Santander, Spain; ^40^ Department of Oncology, Hematology and BMT with Section Pneumology, University Medical Center Hamburg-Eppendorf, Hamburg, Germany; ^41^ Department of Oncology, Geneva University Hospital, Geneva, Switzerland. Current affiliation: Sarah Cannon Research Institute and UCL Cancer Institute, London, United Kingdom; ^42^ Division of Oncology/Hematology, Cantonal Hospital Graubunden, Chur, Switzerland; ^43^ Oncology Institute of Southern Switzerland (IOSI), Bellinzona, Switzerland; ^44^ Universita della Svizzera Italiana, Lugano, Switzerland; ^45^ University of Manchester, Manchester, United Kingdom

## Abstract

The classification of the International Germ-Cell Cancer Collaborative Group (IGCCCG) has been a major advance in the management of germ-cell tumors, but relies on data of only 660 patients with seminoma treated between 1975 and 1990. We re-evaluated this classification in a database from a large international consortium.

**MATERIALS AND METHODS:**

Data on 2,451 men with metastatic seminoma treated with cisplatin- and etoposide-based first-line chemotherapy between 1990 and 2013 were collected from 30 institutions or collaborative groups in Australia, Europe, and North America. Clinical trial and registry data were included. Primary end points were progression-free survival (PFS) and overall survival (OS) calculated from day 1 of treatment. Variables at initial presentation were evaluated for their prognostic impact. Results were validated in an independent validation set of 764 additional patients.

**RESULTS:**

Compared with the initial IGCCCG classification, in our modern series, 5-year PFS improved from 82% to 89% (95% CI, 87 to 90) and 5-year OS from 86% to 95% (95% CI, 94 to 96) in good prognosis, and from 67% to 79% (95% CI, 70 to 85) and 72% to 88% (95% CI, 80 to 93) in intermediate prognosis patients. Lactate dehydrogenase (LDH) proved to be an additional adverse prognostic factor. Good prognosis patients with LDH above 2.5× upper limit of normal had a 3-year PFS of 80% (95% CI, 75 to 84) and a 3-year OS of 92% (95% CI, 88 to 95) versus 92% (95% CI, 90 to 94) and 97% (95% CI, 96 to 98) in the group with lower LDH.

**CONCLUSION:**

PFS and OS in metastatic seminoma significantly improved in our modern series compared with the original data. The original IGCCCG classification retains its relevance, but can be further refined by adding LDH at a cutoff of 2.5× upper limit of normal as an additional adverse prognostic factor.

## INTRODUCTION

About one third of patients with seminoma present with metastatic disease. In 1997, the International Germ-Cell Cancer Collaborative Group (IGCCCG) published a classification, which became the accepted international standard and replaced all previous ones.^1^

**CONTEXT**

**Key Objective**
To reassess the original International Germ-Cell Cancer Collaborative Group (IGCCCG) classification for seminoma with modern treatment data and to screen for additional prognostic variables.
**Knowledge Generated**
Even if applied to modern-type treatments, the IGCCCG classification still separates good prognosis from intermediate prognosis seminoma and retains its relevance for clinical practice. However, survival probabilities have significantly improved in both prognostic groups compared with the original publication from 1997. Lactate dehydrogenase at a cutoff 2.5x the upper limit of normal is identified as a new adverse prognostic variable among otherwise good prognosis patients. Additional variables such as age, extragonadal primary tumor location, human chorionic gonadotropin levels, or the presence of lung metastases were of minor relevance.
**Relevance**
The results of the IGCCCG update analysis help to counsel patients with seminoma more accurately in respect to the treatment outcome they can expect and help to shape future trials in seminoma.

However, the relevance of the original IGCCCG classification has been challenged. According to the original IGCCCG classification, metastatic seminomas are split into good or intermediate prognosis categories based on the presence or absence of liver, bone, or brain metastases.^1^ However, as data from only 660 patients with seminoma had been analyzed, the ability to assess the impact of other relevant variables such as age, lactate dehydrogenase (LDH), elevated human chorionic gonadotropin (HCG) levels, or the presence of pulmonary metastases was limited. Moreover, patients with seminoma included in the original IGCCCG analysis were treated between 1975 and 1990 and many had not received cisplatin or etoposide, which would be the treatment backbone for metastatic seminoma today.^2,3^

The IGCCCG-Update Consortium collected data on metastatic seminoma with two major goals. First, to validate the original IGCCCG criteria and update survival probabilities in a modern cohort. Second, to explore additional prognostic factors that may add granularity to the original IGCCCG prognostic groups and explain some of the heterogeneity found within the prognostic groups of the original IGCCCG classification.

## MATERIALS AND METHODS

### The IGCCCG-Update Consortium

At the time of the first data collection, the IGCCCG-Update Consortium consisted of 30 institutions or collaborative groups in Australia, Europe, and North America. Potential contributors were identified through contact between peers, supplemented by a PubMed search. The principal investigators of individual trials were invited to participate in the initiative based on a written data sharing agreement.

In addition, the coordinators of national cooperative groups in germ-cell tumor (GCT) or principal physicians at large cancer centers were contacted in respect to the availability of national or local cancer registries in electronic format. Investigators were asked to contribute consecutive patients. The Protocol, the contributing centers/groups to the IGCCCG Update Consortium, and the list of collected data items are available in the Data Supplement (online only).

### Data Collection

The purpose of the collaboration was to establish a common electronic database with data of patients with metastatic GCT treated between 1990 and 2013—the IGCCCG-Update Data Warehouse. To ensure appropriate representation of patients, and because trials often limit eligibility to specific IGCCCG prognostic groups, structured data from national registries, databases, or large cohorts of single-center data on consecutively treated patients who fulfilled the protocol data and eligibility requirements were collected in addition to data from clinical trials.

After signing of the data sharing agreements, patient‐level data were aggregated, normalized, and harmonized. Data were processed centrally and stored in a secure format at the headquarters of the European Organisation of Research and Treatment of Cancer in Brussels, Belgium.

### Patients and Data

Thirty participating members of the IGCCCG consortium located in Australia, Europe, and North America provided anonymized, retrospective data on adult male patients with metastatic pure seminoma or retroperitoneal or mediastinal seminoma also when not metastatic.

All patients with seminoma had to receive minimum three cycles of cisplatin- and etoposide-based conventional-dose first-line treatment. Patients who received less than three cycles were also eligible, provided there was enough evidence that at least three cycles were intended. Patients with elevated alpha-fetoprotein level (> 30 ng/mL), with prior chemotherapy for metastatic disease, those included in the original IGCCCG analysis published in 1997 as well as patients with primary GCT of the brain were ineligible. The treatment intended to be given to the patients was recorded where available, otherwise treatments actually given were used.

Data items included the original IGCCCG group, age, date of metastatic diagnosis, primary site, levels of serum alpha-fetoprotein, HCG, and LDH at diagnosis and before chemotherapy as well as the presence and location of metastases. Type and number of chemotherapy cycles were obtained and progression status, vital status, cause of death, and disease status at last follow-up were recorded.

### Trials and Cohorts

We asked for electronic databases of studies and cohorts comprising a minimum of 100 eligible patients for inclusion in the warehouse. Only databases of first-line chemotherapy as described in the patient eligibility criteria were included. Retrospective data of first-line treatments of patients who were primarily referred for relapse were not included because it would have artificially inflated the progression probabilities in the data warehouse.

### Independent Validation

In March 2019, an independent set of patients was collected to validate the prognostic classification. This set consisted of additional patients from the institutions contributing to the analysis set, as well as patients from six new centers located in Australia, Croatia, Spain, Switzerland, and United Kingdom. The eligibility criteria were generally unchanged, but in addition, patients treated between 2013 and March 2016 were allowed, as the outcome data were then mature enough to reach 3 years of expected follow-up. Contributions of less than one hundred patients were accepted for the validation set. Data collection was limited to information regarding original IGCCCG prognostic group, progression-free survival (PFS) and overall survival (OS), treatment regimen as well as LDH and its upper limit of normal (ULN).

### End Points

Primary end points were PFS and OS. OS was defined as the time from start of chemotherapy to death of any cause. PFS was defined from start of chemotherapy to progression, defined by radiologic progression, unequivocal tumor-marker increase, or death, whichever came first. PFS was used for the prognostic model training.

### Statistical Methods

All patients with available PFS and/or OS information were used to update the survival probabilities. Kaplan-Meier estimates were used to update survival estimates according to the original IGCCCG. 95% CIs are provided via log-log transform.^4,5^ Median follow-up estimates for PFS were obtained via the reverse Kaplan-Meier method.^6^

The prognostic model analysis set was formed of patients with all candidate variables available. Candidate variables were the pre-chemotherapy HCG as a continuous variable, LDH levels × upper limit of normal reference range for the laboratory (ULN), site of primary (mediastinum *v* gonadal or retroperitoneal or other), age (in years), and presence of lung metastases. The prognostic model was developed for PFS and applied to both PFS and OS. Both end points were administratively censored at a 3-year horizon to harmonize duration of follow-up across data sources and since most events occurred in this time frame.

Because of the limited number of intermediate prognosis patients available, only the good prognostic group was investigated for additional prognostic factors.

The prognostic model analysis was done via a variable importance algorithm based on conditional survival forest. A conditional survival forest is an ensemble of conditional survival trees aggregated together to improve predictive performance. Each tree is fitted on a random subset of the original analysis set, and only a random subset of the candidate variables can be selected at each binary partitioning. The *variable importance* of each candidate variable is its contribution to the final prediction. The most relevant variables were used to further split the original good prognostic group into subgroups. This analysis was performed using the *ranger* R package.

The discrimination of the new subclassification was assessed using time-dependent area under the receiving curve (AUC) at 3 years.

### Sensitivity Analyses

Not all patients were treated according to international guidelines. To investigate the potential confounding effect of treatment intensity, treatments given were classed into two major clusters: 3 × bleomycin, etoposide, cisplatin (BEP) or 4 × EP (or equivalent), and 4 × BEP (or equivalent). The model was applied to good prognostic patients treated with 3 × BEP or 4 × EP (or equivalent) to assess if model performance is affected by treatment.

All analyses were performed using SAS version 9.4 (Cary, NC) and R software (version 3.6.0).

## RESULTS

### Patient Characteristics

**FIG 1.**
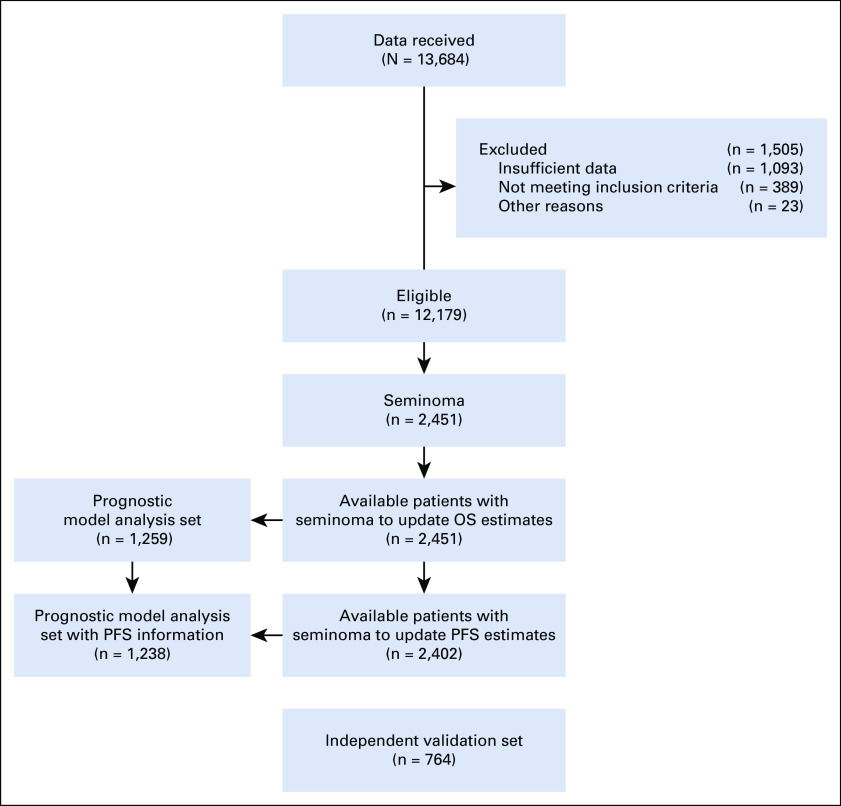
CONSORT diagram. OS, overall survival; PFS, progression-free survival.

In total, data on 13,684 patients with GCT were received, of whom 12,179 (89%) were eligible. The reasons for ineligibility are listed in Figure 1. Of these eligible patients, 2,451 patients had metastatic seminoma and were available for the calculation of OS probabilities. Among them, 2,077 patients were initially recorded in local or national cohorts, whereas 374 came from clinical trial databases. Because of inconsistent or missing progression data, only 2,402 out of 2,451 (98%) patients were used to update PFS probabilities.

Disease progression occurred in 304 patients, and 140 patients died. The median follow-up for PFS was 6.1 years (6.6 year for cohorts, 2.8 for trials) and 81% had been followed for at least 3 years from start of chemotherapy (87% for cohorts, 45% for trials). The median follow-up among survivors was 5.9 years, and the said follow-up ranged from 18 days to 26 years.

The prognostic model analysis set was restricted to 1,259 patients (1,197 good prognosis and 62 intermediate prognosis) with all candidate variables available, of which 1,238 (1,176 good prognosis and 62 intermediate prognosis) also had information on date of progression.

**FIG A1.**
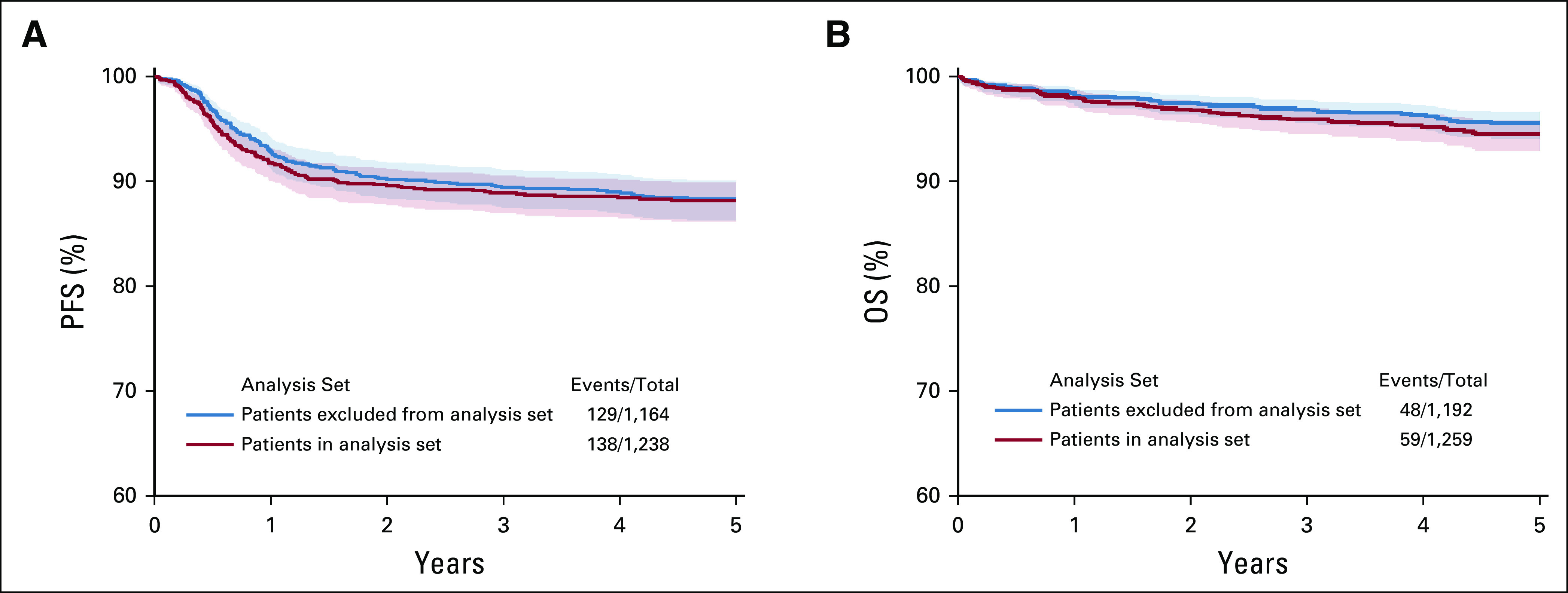
Survival probabilities and 95% CIs for patients in or out of the analysis set for (A) PFS and (B) OS. OS, overall survival; PFS, progression-free survival.

Appendix Table A1 (online only), shows the baseline characteristics of the 2,451 patients overall and divided between patients included or not included in the prognostic model analysis set because of missing data, showing no marked difference between them; however, more cohort patients (92.7%) were excluded from the analysis set compared with trial patients. The most common reason for exclusion was incomplete information about LDH (70% of excluded patients). PFS and OS Kaplan-Meier curves confirm absence of selection bias (Appendix Fig A1, online only).

### Updated Outcomes by Original IGCCCG

**FIG 2.**
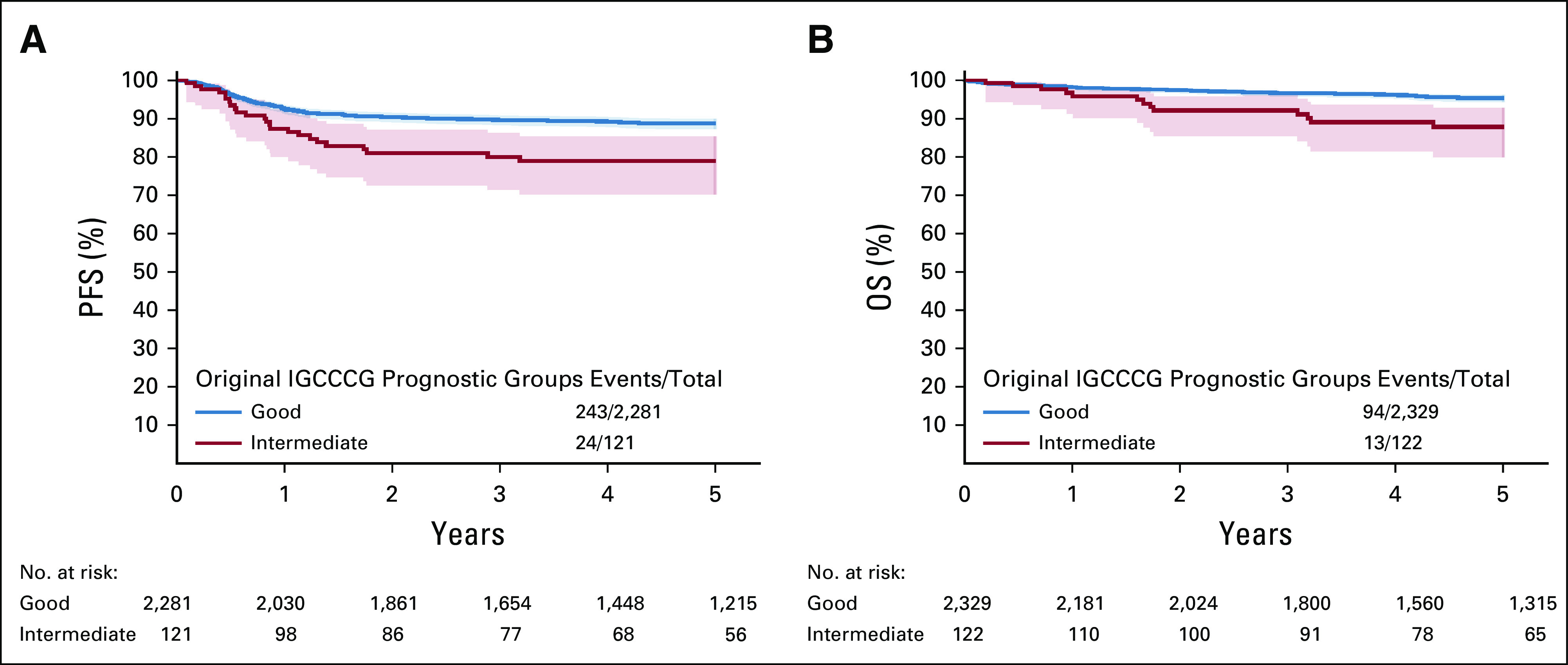
Survival probabilities and 95% CI according to original IGCCCG prognostic groups for (A) PFS and (B) OS. IGCCCG, International Germ-Cell Cancer Collaborative Group; OS, overall survival; PFS, progression-free survival.

The 5-year PFS probability was 89% (95% CI, 87 to 90) and 79% (95% CI, 70 to 85) in good and intermediate IGCCCG prognostic groups, respectively (Fig 2A). The corresponding figures in the 1997 publication were 82% and 67%.

The 5-year OS probability was 95% (95% CI, 94 to 96) and 88% (95% CI, 80 to 93) in good and intermediate prognostic groups, respectively (Fig 2B). The corresponding figures in the 1997 publication were 86% and 72%.

Formal statistical comparisons were not possible since the CI were not reported in the original publication.

### New Prognostic Factors

**FIG A2.**
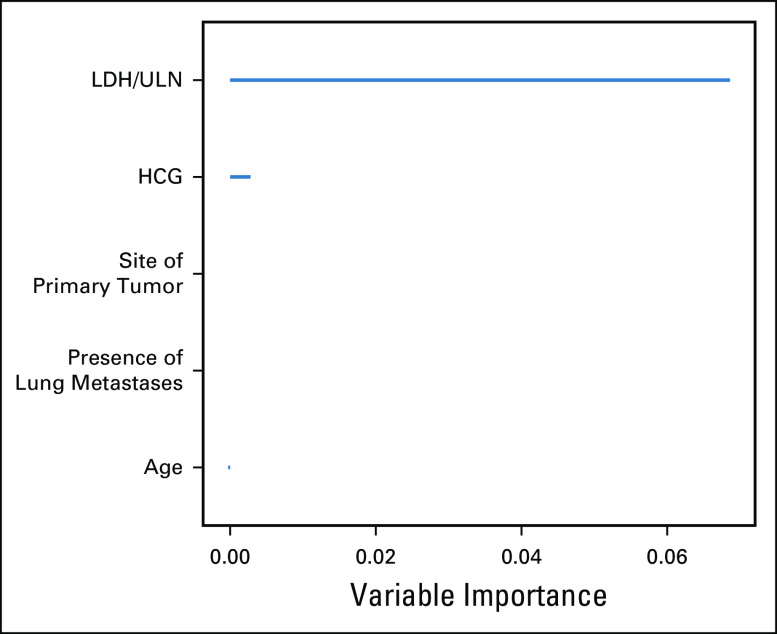
Variable importance of all candidate variables on PFS in the prognostic model analysis set. HCG, human chorionic gonadotropin; LDH, lactate dehydrogenase; ULN, upper limit of normal.

A total of 1,176 good prognosis patients with PFS information were used for training. Appendix Figure A2 (online only). shows the result of the variable importance algorithm. LDH standardized by its ULN was identified as the single most significant prognostic factor for PFS in good prognosis patients. In addition, hCG appears to be weakly informative to predict patient prognosis, but its relative importance is marginal compared with LDH divided by UNL (25 times less). No other variable appeared relevant (Data Supplement).

To select the optimal threshold for LDH/ULN, 20,000 conditional survival trees were fitted on randomly drawn subset of the analysis set, and the threshold chosen was collected for each tree. Thresholds of 2× and 2.5× ULN of LDH were most often proposed by the conditional trees. The threshold of 2.5× ULN was chosen because of its higher specificity; 267 out of 1,197 good prognosis patients (22.3%) had LDH above 2.5× ULN.

**FIG 3.**
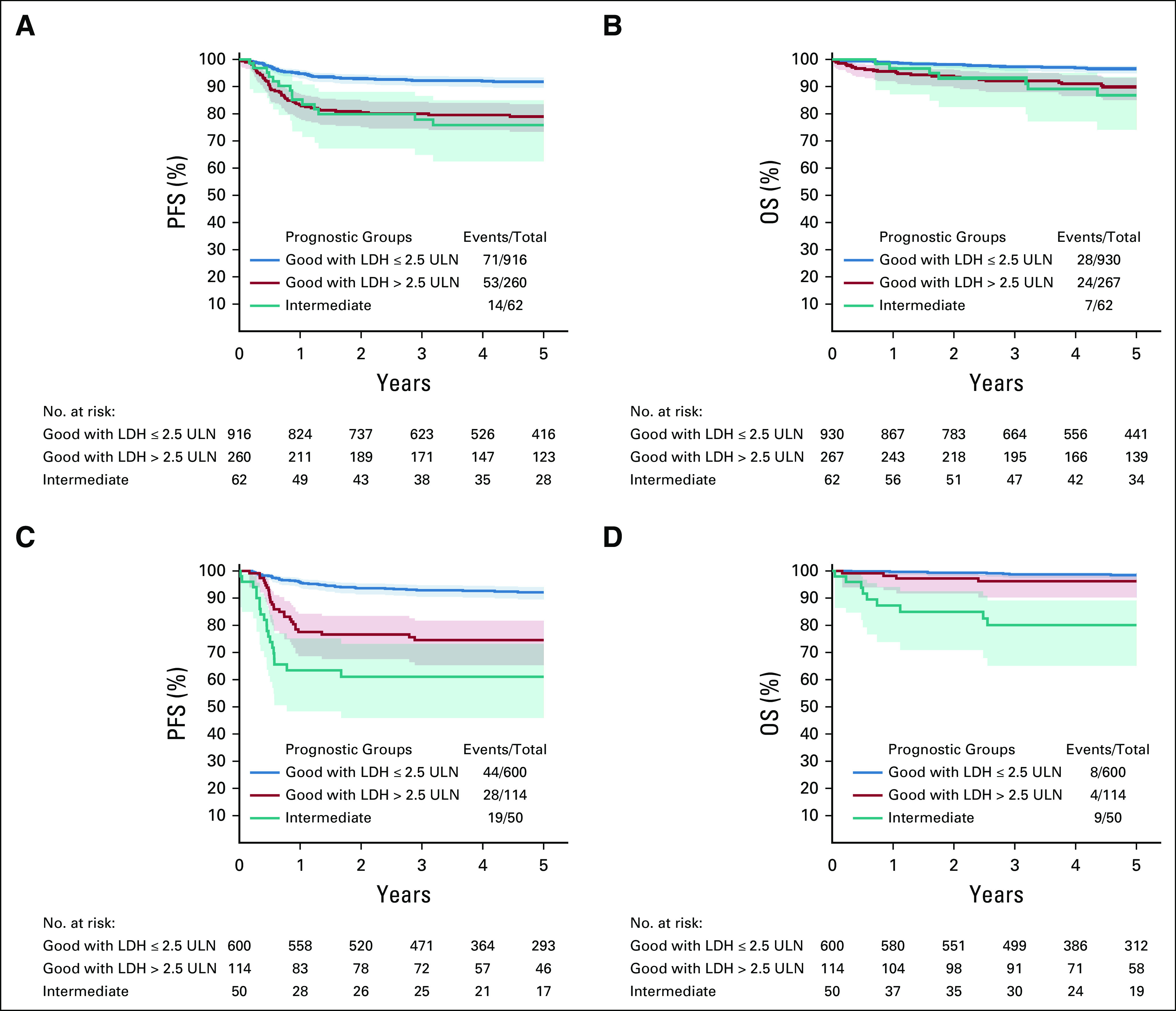
(A) PFS and (B) OS probabilities and 95% CI in the analysis set and (C, D) in the validation set by original International Germ-Cell Cancer Collaborative Group prognostic group with subdivision of good risk by LDH level (≤ 2.5 ULN, > 2.5 ULN). LDH, lactate dehydrogenase; OS, overall survival; PFS, progression-free survival; ULN, upper limit of normal.

Figure 3A shows PFS and OS in the analysis set for this classification. Good prognostic IGCCCG patients with LDH above 2.5× ULN had worse survival probabilities than patients with LDH below or equal to 2.5× ULN, as shown in Table 1. Good prognosis patients with LDH above 2.5× ULN had a 3-year PFS of only 80% (95% CI, 75 to 84) and a 3-year OS of only 92% (95% CI, 88 to 95) versus 92% (95% CI, 90 to 94) and 97% (95% CI, 96 to 98) in the group with lower LDH. In particular, the survival probabilities of good prognosis patients with an LDH above 2.5× ULN were similar to the intermediate prognosis patients. This was true for both end points, although the differences are less striking for OS because of the low number of deaths observed. In the analysis set, the time-dependent AUC at 3 years was 0.61.

**TABLE 1.**
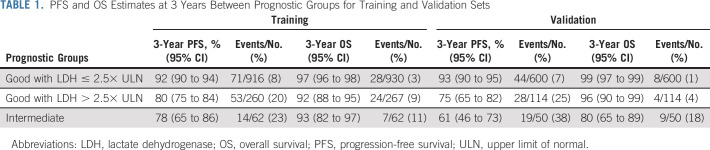
PFS and OS Estimates at 3 Years Between Prognostic Groups for Training and Validation Sets

### Independent Validation

To validate LDH as a new prognostic factor, 764 patients were independently collected. The set consisted of 554 additional patients treated between 1995 and 2016 at the institutions contributing to the analysis set, and 210 patients treated between 1996 and 2016 from the six additional centers.

Overall, 114 out of 714 good prognosis patients (15.9%) had an LDH above 2.5× ULN. As shown in Figure 3B and Table 1, the difference in PFS observed between the two subgroups of good prognosis patients is maintained, as well as the discriminative ability, with a 3-year AUC of 0.64. This difference seems to hold for OS as well, but this cannot be tested owing to small number of events.

### Sensitivity Analysis on Treatment Intensity

Table 2 shows the repartition of patients forming the training and validation sets treated with 3 × BEP or 4 × EP (or equivalent) versus 4 × BEP (or equivalent). 4 × BEP (or equivalent) contains patients treated with 4 × BEP, 4 × vincristine, ifosfamide, cisplatin, 4 × paclitaxel, bleomycin, etoposide, cisplatin (TBEP), and carboplatin, bleomycin, vincristine, cisplatin (CBOP)/BEP.

**TABLE 2.**
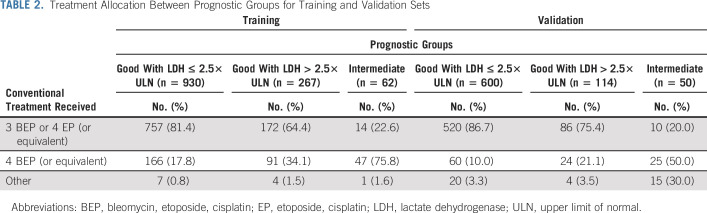
Treatment Allocation Between Prognostic Groups for Training and Validation Sets

**FIG 4.**
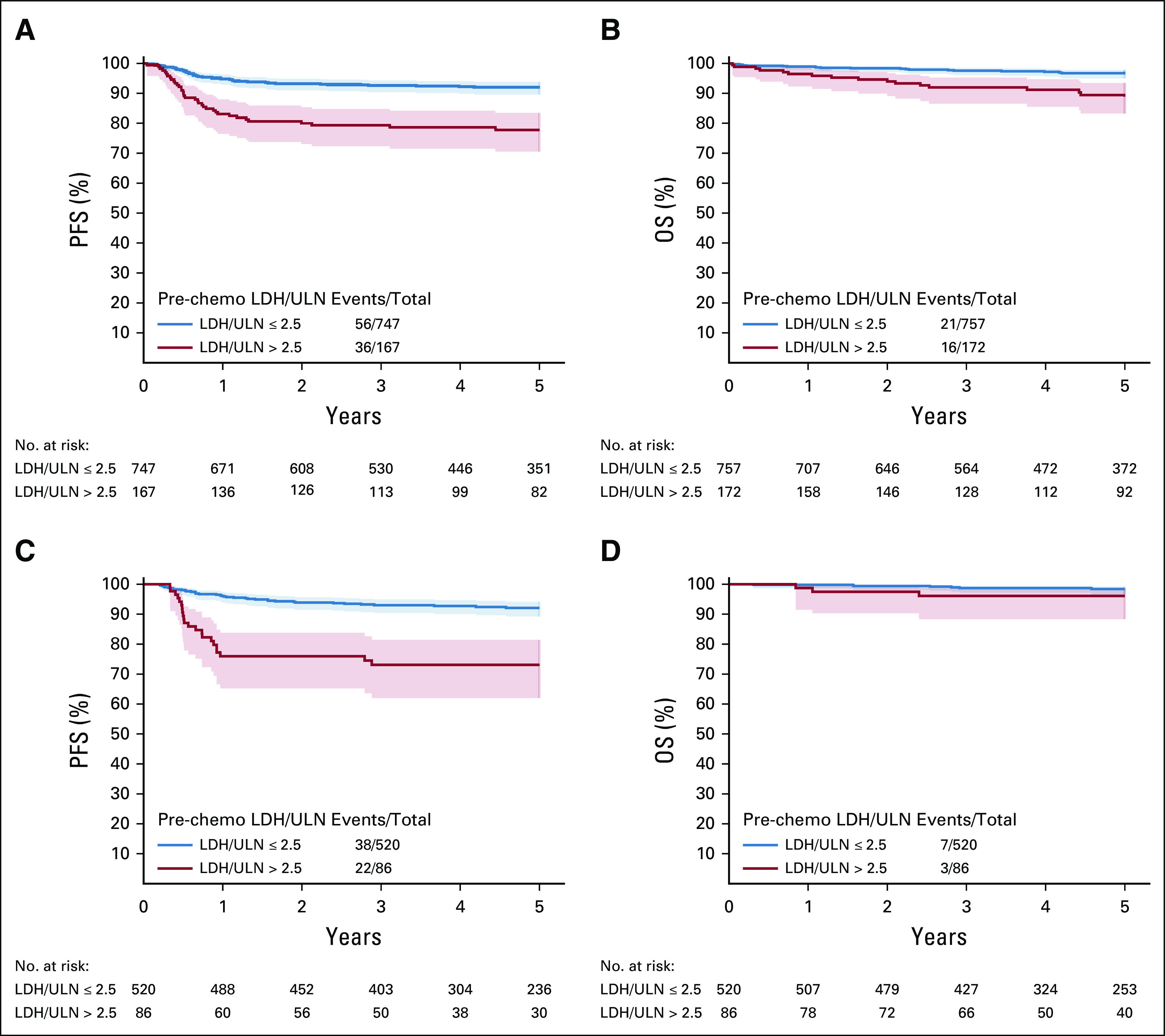
Survival probabilities and 95% CI of good prognosis patients according to LDH levels in the analysis set for (A) PFS and (B) OS, as well as in the (C, D) validation set, restricted to patients treated with three BEP or four EP. BEP, bleomycin, etoposide, cisplatin; EP, etoposide, cisplatin; LDH, lactate dehydrogenase; OS, overall survival; PFS, progression-free survival; ULN, upper limit of normal.

Figure 4 shows the PFS according to the new classification among good prognosis patients who were treated according to international guidelines, with 3 × BEP or 4 × EP. When restricting to patients treated with 3 × BEP or 4 × EP, the difference in PFS between low and high LDH in good prognosis patients remained, in both the training set and the validation set, excluding that the difference might be because of different treatment approaches. The time-dependent AUC at 3 years was 0.61 in the restricted training set and 0.64 in the restricted validation set.

Similar findings were also observed for OS in the analysis set; however, this was based on fewer events.

## DISCUSSION

The international IGCCCG-Update Consortium database is the largest source of information on metastatic GCT worldwide. In this report, we analyzed the subset of 2,451 patients with metastatic seminoma treated between 1990 and 2013 with modern-type cisplatin- and etoposide-based chemotherapy and validated our results in additional 764 patients.

Compared with the results of the original IGCCCG cohort, which were based on data on only 660 patients with seminoma, good prognosis as well as intermediate prognosis metastatic seminoma in the IGCCCG-Update Consortium database experienced substantially improved survival. Previous smaller series in patients with GCT also reported better results compared with the original IGCCCG cohort.^7-11^ However, the IGCCCG database is multicenter, international, and by far the largest collection for patients with pure seminoma. Improvements may have resulted from stage migration because of earlier diagnosis and better diagnostic tools, improved supportive care, superiority of cisplatin- and etoposide-based first-line treatment over other conventional combinations, the use of upfront dose-intensified regimens, more stringent use and higher quality of post-chemotherapy management, better salvage strategies in nonresponding or relapsing patients, more stringent guideline adherence, centralization of care at experienced expert centers, or a combination of these factors. Given 5-year PFS and OS survival probabilities of 88% (95% CI, 87 to 89) and 95% (95% CI, 94 to 96), respectively, across all prognostic groups, metastatic seminoma represents the most curable metastatic cancer in males.

An important finding of the present analysis is that the original IGCCCG classification as published in 1997 still correctly identifies two prognostic groups with significantly different PFS and OS probabilities. However, we identified LDH above 2.5× ULN levels before chemotherapy as an additional adverse prognostic factor that allowed splitting the good prognostic group further. Good prognosis patients with an elevated LDH above 2.5× ULN before chemotherapy experienced significantly worse outcomes. This finding confirms previous reports that suggested elevated LDH as an adverse prognostic factor in metastatic seminoma, but in much smaller sample sizes.^9,12-14^ In the original IGCCCG publication, LDH was also identified as an adverse prognostic factor with a cutoff of 2× ULN, but was not added to the final classification. However, in this analysis, we suggest a cutoff of 2.5× ULN because of its higher specificity.

Other variables such as age, extragonadal primary tumor, elevated levels of hCG, or the presence of lung metastases did not add significant prognostic information in good prognosis patients once LDH elevations were considered.

The majority of patients in the IGCCCG-Update Consortium database were treated according to international guidelines consisting of three cycles BEP or four cycles EP in good prognosis patients and four cycles BEP or three cycles BEP plus one cycle EP in intermediate prognosis patients. Interestingly, in both the training and validation sets, patients with elevated LDH were more often treated with four cycles of BEP or equivalent, compared with good patients with low LDH (34.1% *v* 17.8% in analysis set, and 21.1% *v* 10% in validation set). Possibly, based on the reports mentioned, some experts had already stepped away from international guidelines and intensified treatment for good prognosis patients with elevated LDH.

However, because of its retrospective nature, our analysis cannot inform which treatment strategy is best in any of the described subsets. Until the availability of prospective trials results, our analysis does not allow to recommend, for example, using four instead of three cycles BEP in good (according to the original classification) prognosis patients who have an LDH level above 2.5× ULN. Additionally, despite the large international effort, the sample size of intermediate prognosis seminoma in the IGCCCG-Update Consortium database was too small to study any additional prognostic factors.

Because of these limitations, the original IGCCCG classification as published in 1997 remains the reference for treatment decisions in daily practice as all currently available trials used the original IGCCCG classification for treatment stratification. However, refinement of the original IGCCCG classification by adding LDH as an adverse prognostic factor within the good prognostic group allows for a better assessment of individual patients and can help to shape future treatment strategies in metastatic seminoma. Knowledge about the prognostic impact of LDH supports the development of trials with de-escalation strategies in good prognosis seminoma with low LDH to further reduce treatment burden in patients likely to be cured. By contrast, inferior outcomes despite modern-type cisplatin- and etoposide-based chemotherapy in good prognosis seminoma with elevated LDH as well as in intermediate prognosis seminoma underscore the need to re-evaluate and improve current treatment strategies in such patients in prospective clinical trials.

In conclusion, we confirm that the original IGCCCG classification retains its relevance in metastatic seminoma, but with clearly improved outcomes. The classification can be further refined by adding LDH at a cutoff of 2.5× ULN as an additional adverse prognostic factor within the good prognosis group.

## Figures and Tables

**TABLE A1.**
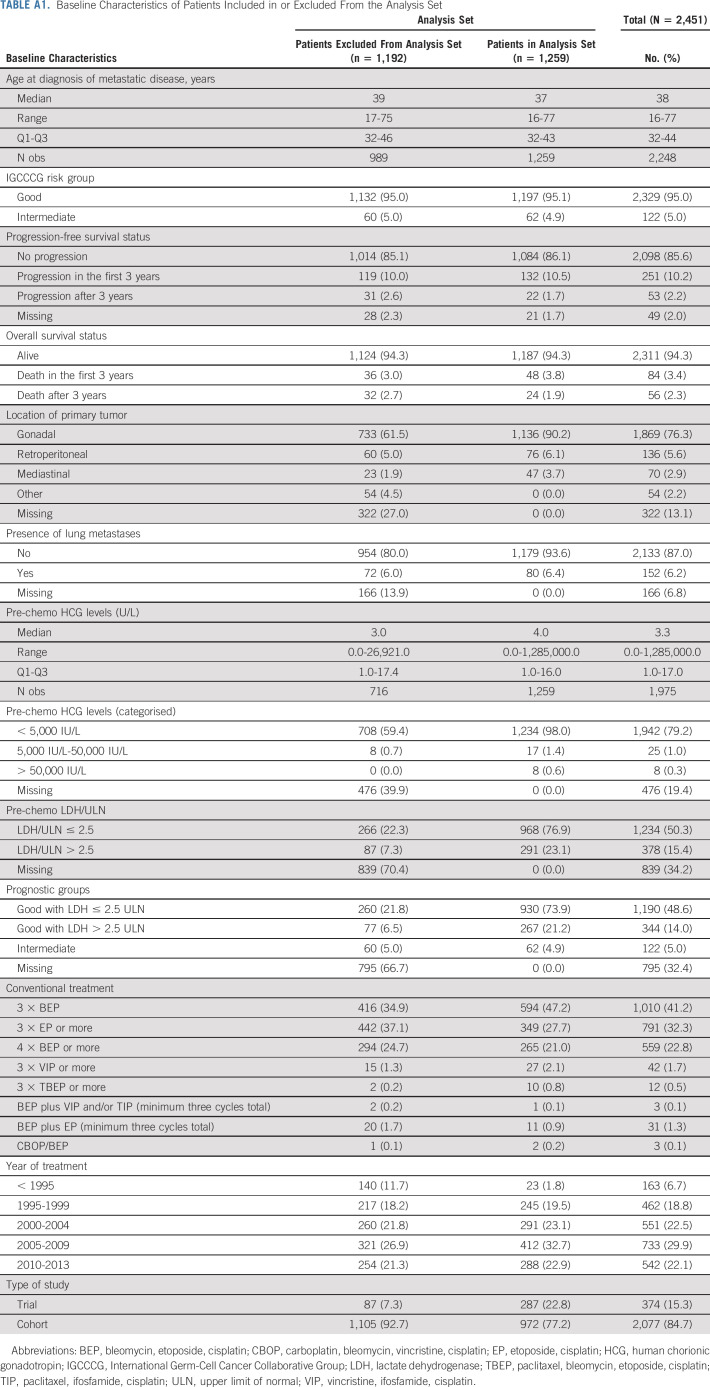
Baseline Characteristics of Patients Included in or Excluded From the Analysis Set

## APPENDIX International Germ-Cell Cancer Classification Update Consortium

We thank the following centers, research groups, and cancer registries who contributed data and supported the IGCCCG-Update Consortium:

### Contributors to the IGCCCG-Update Consortium Data Warehouse

#### Australia.

Australian and New Zealand Urogenital and Prostate (ANZUP) Cancer Trials Group, Sydney and investigators of the P3BEP study (ACTRN12613000496718), and ANZUP Good prognosis GCT study (ACTRN12605000142639) trials.

#### Canada.

Princess Margaret Cancer Centre, University Health Network, Toronto, Ontario; Indiana Tom Baker Cancer Centre, University of Calgary, Calgary, Alberta; Western University and London Health Sciences Centre, London, Ontario.

### Europe

#### Belgium.

The European Organisation for Research and Treatment of Cancer (EORTC) Genito-Urinary Cancer Group and investigators of the EORTC 30895, EORTC 30941, EORTC 30974, and EORTC 30983 trials; EORTC Headquarters, Brussels.

#### Austria.

Medical University of Graz.

#### Denmark.

Righshopitalet Copenhagen and contributors to the Danish Germ Cell Cancer Registry.

#### France.

Institut Gustave Roussy, University of Paris Saclay, Villejuif; Groupe d’Etude des Tumeurs Uro-Génitale (GETUG) and investigators of the GETUG-13, S99, and T93 trials.

#### Germany.

Universitätsklinikum Düsseldorf, Düsseldorf; Universitätsklinikum Köln/Köln University Hospital, Köln; Red Cross Hospital, University of Munich, Munich; University Medical Center Hamburg-Eppendorf, Hamburg; the German Testicular Cancer Study Group and investigators of the HDVIP protocol.

#### Italy.

San Camillo Forlanini Hospital, Rome; Fondazione IRCCS Istituto Nazionale dei Tumori, Milano; the Italian Germ cell Cancer Group (IGG).

#### Norway and Sweden.

The Norwegian University of Science and Technology, Trondheim, Norway; Oslo University Hospital, Oslo, Norway; Skåne University Hospital, Lund, Sweden; The Swedish and Norwegian Testicular Cancer Group (SWENOTECA).

#### Slovakia.

National Cancer Institute, Bratislava.

#### Spain.

Hospital Universitario Morales Meseguer-IMIB, Murcia; Universidad Católica San Antonio de Murcia (UCAM), Murcia; Institut Catala d'Oncologia, Barcelona; the Spanish Germ Cell Cancer Group (SGCCG) Group and contributors to the SGCCG registry and of the “chemotherapy versus radiotherapy study” (DOI: 10.1200/JCO.2007.15.9103).

#### Switzerland.

University of Zürich, Zürich.

#### The Netherlands.

University Medical Center Groningen, Groningen.

#### The United Kingdom.

The Institute of Cancer Research, Sutton; the Medical Research Council Testicular Cancer Study Group and investigators of the MRC TE13 and TE20 studies; St Bartholomew’s Hospital, London.

#### Russia.

N.N. Blokhin Russian Cancer Research Center, Moscow, Russian Federation.

#### The United States of America.

Indiana University, Melvin and Bren Simon Cancer Center, Indianapolis, Indiana; Dana-Farber Cancer Institute—Harvard Medical School, Boston, MA; University of Pennsylvania, Perelman Center for Advanced Medicine, Philadelphia, PA; Memorial Sloan Kettering Cancer Center, New York, NY, and investigators of the MSKCC0747 and MSKCC94076 studies.

### IGCCCG-Update Supporting Centers That Contributed to the Seminoma Validation Set

Peter MacCallum Cancer Centre, Melbourne, Australia

University Hospital Center Zagreb, Zagreb, Croatia

Hospital Universitario Marques de Valdecilla and IDIVAL, Santander, Spain

Division of Oncology/Hematology, Cantonal Hospital Graubünden, Chur, Switzerland

Geneva University Hospital, Geneva, Switzerland

Leeds Institute of Medical Research, University of Leeds, Leeds, UK
